# A novel intelligent photonic design method enabled by metamaterials and k-nearest neighbor

**DOI:** 10.1515/nanoph-2024-0409

**Published:** 2025-01-06

**Authors:** Hangming Fan, Junlin Pan, Yongchen Wang, Zhe Yuan, Mengfan Cheng, Qi Yang, Deming Liu, Lei Deng

**Affiliations:** Wuhan National Laboratory for Optoelectronics and School of Optical and Electronic Information, 12443 Huazhong University of Science and Technology, Wuhan 430074, China; Shenzhen Huazhong University of Science and Technology Research Institute, Shenzhen 518000, China; JinYinHu Laboratory, Wuhan 430040, China

**Keywords:** metamaterial, machine learning, inverse design, power splitter

## Abstract

The utilization of metamaterials plays a pivotal role in integrated photonics. The precise design of metamaterials enables them to finely manipulate light, resulting in an ultra-compact footprint and exceptional performance that cannot be achieved by traditional structures. The conventional methods for metamaterial design, however, encounter challenges from intricate targets. Although attempts have been made to apply inverse design to metamaterials, there is still a need for a highly intelligent, low-computation method, and easy-to-fabricate metamaterial structure. Here, we present an efficient methodology that combines metamaterials, heuristic algorithms, and machine learning to facilitate the rapid development of intricate devices. The method is used to design 1 × N power splitters with arbitrary power ratios, as an application example. Specifically, 1 × 2, 1 × 3, 1 × 4 power splitters with arbitrary ratios are fabricated and experimentally demonstrated. The application of this method in arbitrary power splitter highlights its appropriateness for the design and optimization within integrated photonics devices.

## 1 Introduction

In recent years, there has been significant advancement in the field of integrated photonics. Metamaterials have emerged as a crucial tool in the design of integrated photonics due to their extensive design flexibility and precise manipulation of light fields [1]. These advantages make metamaterials widely used in silicon devices such as mode-division multiplexers [2], wavelength multiplexers [3], mode-size converters [4], and so on. The conventional approach to metamaterial design heavily relies on the designer’s expertise, and the footprint will significantly increase as the complexity grows. The inverse design [5] offers a viable solution to achieve intricate structures and an exceptionally compact footprint, surpassing the capabilities of conventional methods.

The process of inverse design involves transforming device design into a systematic approach for identifying the optimal solution for the structure. Once the target is set, inverse design methods automatically determine the most suitable structural parameters or patterns. As a result, algorithms enable the effortless acquisition of intricate structures that are otherwise challenging to manually conceive.

In order to fully exploit the flexibility of metamaterials, many structural types have been proposed, such as empirical structures [6], QR-code-like structures [7], and irregular structures [8]. To solve the black-box problem of different structure types, many different optimization algorithms are proposed, such as heuristic algorithms [9], [10], brute-force searching algorithms [7], and gradient-based algorithms (e.g., gradient descent optimization [8] and topology optimization [11]). However, the irregular structures often possess feature sizes that are too small to be fabricated. Empirical structures offer limited design freedom. Design algorithms resembling QR codes require significant time and are highly influenced by the initial graphics. The majority of inverse design methods require recalculation when there is even a slight change in the figure of merit (FOM). Recently, significant advancements have been made in photonic inverse design through the application of artificial intelligence (AI). Among these methods, artificial neural networks (ANN) have emerged as a pivotal approach for efficiently solving inverse design problems compared to traditional optimization algorithms. Moreover, a well-trained ANN can be utilized iteratively to devise devices with varying FOM while maintaining the same structural foundation [12]. The dataset for training artificial neural networks (ANN) plays a crucial role in achieving optimal network performance, as it necessitates a substantial amount of training data. For instance, in Ref.12, approximately 20,000 samples were utilized solely for the design of a 1 × 2 power splitter with arbitrary power ratios. Despite this, the computational cost associated with ANN remains significant. Although there are many new network structures (such as tandem network (TNN) [13]) that can be well applied to this type of problem, the large amount of data is still the main problem of neural network methods. Consequently, there is still an evident absence of a metamaterial inverse design method that combines exceptional manufacturability and high computational efficiency.

In this paper, we propose an intelligent inverse design method for metamaterials. By utilizing subwavelength gratings as the structural foundation and integrating heuristic algorithm particle swarm optimization (PSO) with the machine learning technique K-Nearest Neighbor (KNN), a novel inverse design structure is achieved, characterized by high design flexibility, low computational complexity, small footprint, and excellent manufacturability. The structures can be strategically integrated into any position to achieve intricate functionalities or enhance the performance of pre-existing devices. The efficiency and scalability of this method are demonstrated by designing a 1 × N power splitter with an arbitrary power ratio. The attractiveness of splitters with arbitrary output port numbers and arbitrary power ratios surpasses that of power splitters with uniform split ratios due to their wider range of applications such as feedback circuits [14], optimal power distribution [15], tap-port power monitoring, and so on. Recently, a variety of 1 × N (N>2) splitters have been reported. Traditional structures such as multimode interference MMI [16], Y-branches [17], and photonic crystal [18] are typically fixed with limited parameters for manipulation, posing challenges in achieving arbitrary power ratios through design. The work in [19] proposed a 1 × N power splitter with arbitrary power ratios based on a directional coupler; however, the design heavily relies on the expertise of the designer and results in a significant expansion of footprint as the number of output ports increases. In this study, we have designed 1 × 2 power splitters with ratios of 1:1, 2:1, 6:4, and 9:1, 1 × 3 power splitters with ratios of 1:1:1, and 1:2:1, and 1 × 4 power splitters with ratios of 1:1:1:1, 3:2:2:3, and 4:3:2:1. All the inverse design regions possess a compact footprint, measuring less than 4 μm × 2.6 μm. Additionally, only a limited number of data samples are utilized for each specific number of output ports. Finally, we have successfully fabricated the chip and experimentally demonstrated that the structures are highly compatible with our fabrication process. The insertion loss of all splitters at 1550 nm is consistently below 1 dB, while the power ratio difference remains within a narrow range of 1.5 dB. Additionally, the scanning electron microscope (SEM) images exhibit exceptional fabrication accuracy, thus validating the effectiveness and feasibility of our intelligent metamaterials (IM) method.

## 2 Design and simulation

### 2.1 Design principle

**Figure 1:**
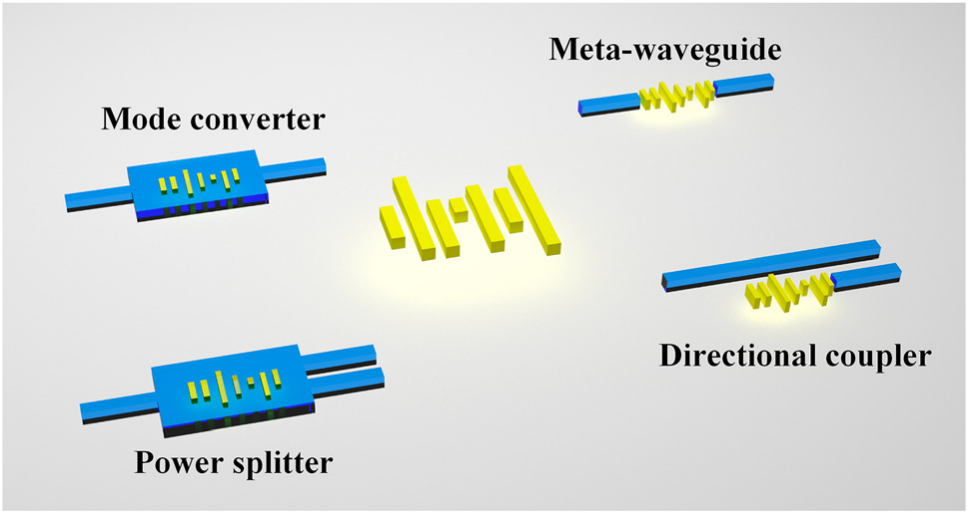
The schematic of the intelligent metamaterial, and the potential devices.

**Figure 2:**
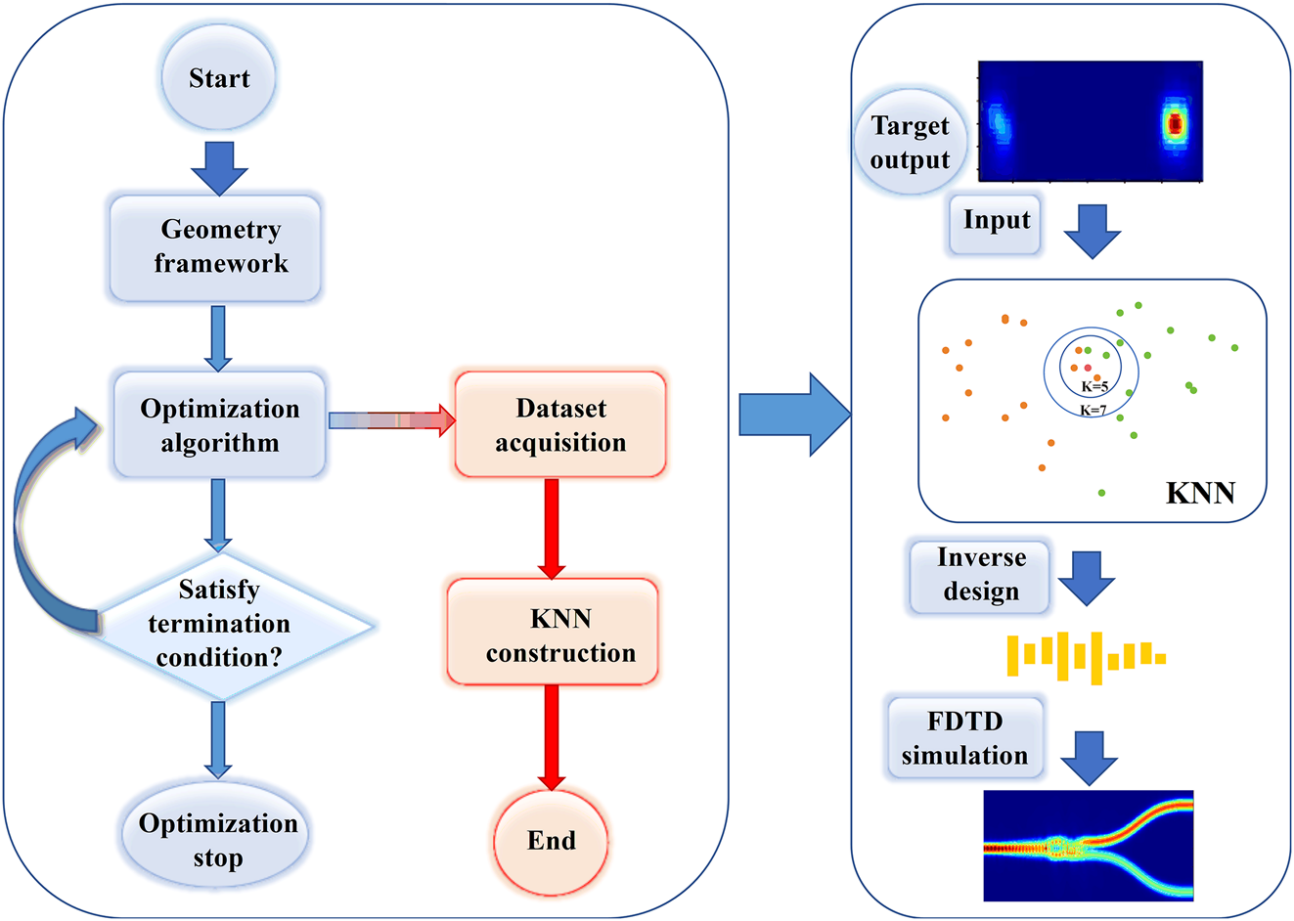
The flow chart of the intelligent metamaterial design.

Figure 1 shows the schematic structure diagram of the proposed IM. The structure consists of a subwavelength rectangular array, with each rectangle having three independent design parameters: length, width, and position. The N rectangles owned by IM possess 3 N design dimensions, enabling the support of complex functions. IM can be flexibly integrated into structures as grooves or waveguides, as illustrated in Figure 1. This allows for the potential realization or optimization of various devices through IM, including mode conversion, power splitting, meta-waveguide implementation, and directional coupling. The flow chart of the intelligent metamaterial design is illustrated in Figure 2, comprising two distinct stages. The KNN construction process, Stage 1, involves optimizing the IM using an optimization algorithm to obtain the dataset. Subsequently, the KNN can be constructed based on this dataset. The second stage involves the inverse design process. The target output of the structure is utilized as input for KNN, resulting in obtaining the 3 N parameters of IM. Subsequently, the structure undergoes verification through FDTD simulation.

The most important step of KNN is using the K-dimensional tree (Kd-tree) to find the K values of the sample’s nearest neighbors. Kd-tree is a kind of data structure that divides K-dimensional data space. Each node of a Kd-tree is a binary tree of K-dimensional points. All non-leaf nodes can be viewed as hyperplanes that divide a space into two half-spaces. The subtree to the left of the node represents the point to the left of the hyperplane (the set of points smaller than the hyperplane in the split dimension), and the subtree to the right of the node represents the point to the right of the hyperplane (the set of points larger than the hyperplane in the split dimension). When the Kd-tree is implemented, it mainly includes two parts: 1. Creation of Kd-tree; 2. Nearest neighbor search.

1. Creation of Kd-tree: select one dimension with the maximum variance in the K-dimensional data set, the dimension *K* here is the number of outputs, and the variance refers to:

$$S_{K}=\overset{m}{\underset{i=1}{\sum }}{\left|P_{i}-P\right|}^{2}$$

Where *m* refers to the number of data, *P*_i_ refers to the output of each data, and *P* refers to the average output of all data. Then select the median value in this dimension as a pivot to divide the data set, and obtain two sub-sets; Repeat the process of the last step for two subsets until all subsets can no longer be divided.

2. Nearest neighbor search: Here we use Euclidean distance(*d*) to represent distance:

$$d=\sqrt{\overset{K}{\underset{n=1}{\sum }}{\left|P_{n}-T_{n}\right|}^{2}}$$

*P*_n_ represents the actual output, and *T*_n_ refers to the target output. A backtracking operation is performed to find the “nearest neighbor point” closer to *T*_n_. That is, to determine whether any points in the unvisited branch are closer to *T*_n_, and the distance between them is less than *d*. If the distance between Tn and the unvisited branch under its parent node is less than *d*, it is considered that there is data closer to *T*_n_ in this branch. The search process as in the last step is carried out when entering this node. If the nearest data point is found, it is updated to the nearest point and *d* is updated. If the distance between *T*_n_  and the unvisited branch under its parent is greater than *d*, then there is no point in the branch closer to *T*_n_. Repeat this step *k* times to find the *k* points closest to *T*_n_, and average these *k* points to get the parameters of the target structure.

**Figure 3:**
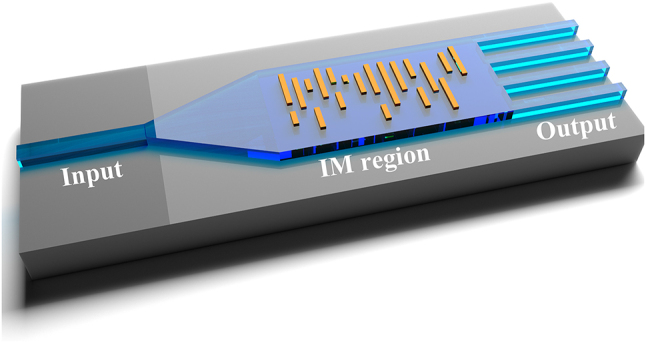
The schematic of the power splitter with arbitrary power ratio and output port numbers is based on the proposed intelligent metamaterial.

As an illustration of the IM design methodology, we have developed 1 × N power splitters with customizable power ratios. The schematic structure diagram of the power splitter based on intelligent metamaterial is illustrated in Figure 3. It comprises an input port, a taper that offers ample design flexibility for inverse design, an intelligent metamaterial region, and output ports. The IM structure is configured with a uniform d value equal to 50 % of the grating period. The power ratio in the dataset is optimized using particle swarm optimization (PSO) as a first step. In PSO, the figure of merit (FOM) is defined as:

$$\text{FOM}=\text{abs}\left(T_{1}-P_{1}\right)+\text{abs}\left(T_{2}-P_{2}\right)+\cdots +\text{abs}\left(T_{N}-P_{N}\right)$$

Where the P_N_ refers to the power of output port N, and T_N_ refers to the target output ratio. The fitness of all particles is calculated in each generation within the particle swarm algorithm, where the positions of the particles represent the structural parameters and the fitness is determined by evaluating power ratios. Consequently, after each calculation iteration, we store both the positions of all particles and their corresponding power ratios to establish a relationship between structure and power ratio. The parameter sets and power ratios of all particles are saved as a dataset for KNN after each iteration in the PSO process. This dataset is then divided into two parts: 80 % is utilized for KNN model prediction, while the remaining 20 % serves as test data for performance evaluation. The evaluation method employed in this study is root mean square error (RMSE):

$$\text{RMSE}=\sqrt{\frac{1}{n}\overset{n}{\underset{i=1}{\sum }}{\left(h_{i}-h_{p}\right)}^{2}}$$

**Figure 4:**
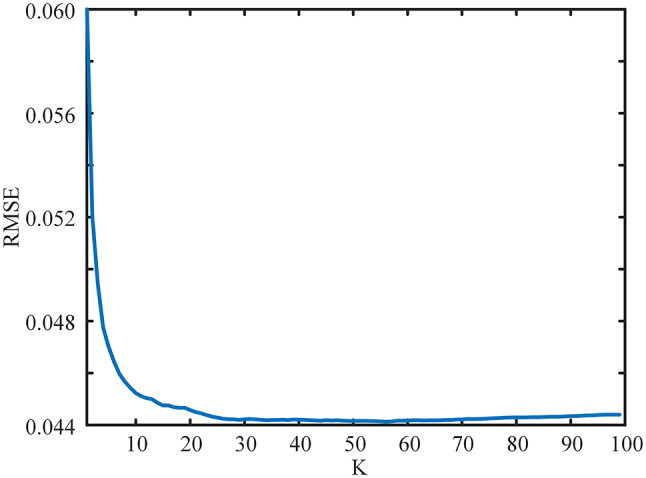
The variation of RMSE with K.

Where *h*_*i*_ and *h*_*p*_ are the predicted parameters and the accurate parameters, respectively. The key parameters in KNN include the selection of the k-value. We used the cross-validation method to compute the effect on the results when K is taken at different values and obtained Figure 4. It can be seen that the RMSE decreases rapidly when K increases, and starts to increase when K is greater than 60. Because the value of K is generally not more than 20 and is an odd number, after substituting the actual verification of the effect of different values of K on the results, the overall consideration is to choose the value of K to be 15.

After the setup of KNN is completed, we input the power ratio that requires prediction into KNN to obtain the corresponding metamaterial structural parameters. Finally, FDTD simulation is employed to validate the performance of the inverse design structures. The insertion loss and power ratio are utilized to showcase the performance of the splitters, with their definitions as follows:

$$\text{insertion}\;\text{loss}=-10log\left[\left(P_{1}+\cdots +P_{N}\right)/P_{in}\right]$$

$$\text{power}\;\text{ratio}\left(\text{P}{\text{r}}_{\text{M}}\right)=P_{M}/\left(P_{1}+\cdots +P_{N}\right)$$

The term *P*_*M*_ represents the power of output port *M*. The utilization of the calculation complex enables this method to generate diverse structures with varying power ratios. The required computational complexity of this scheme is significantly reduced compared to traditional methods as the number of design power ratios increases. The traditional optimization approach requires m calculations for one ratio, while *n* kinds of ratios require *n* × m calculations. In this process, m calculations represent the number of simulations conducted by commercial software during the optimization process, which in particle swarm optimization algorithm is “iterations × number of particles”. In contrast, for the IM method, PSO algorithm is run only once to obtain the dataset, and different power ratios are obtained by KNN. Thus it only requires m calculations to obtain *n* kinds of ratios. The amount of training data is reduced by an order of magnitude compared to ANN. The feature size of the metamaterial can be easily controlled by setting the upper and lower limits of parameters, facilitating its fabrication.

### 2.2 1 × 2 power splitters

**Figure 5:**
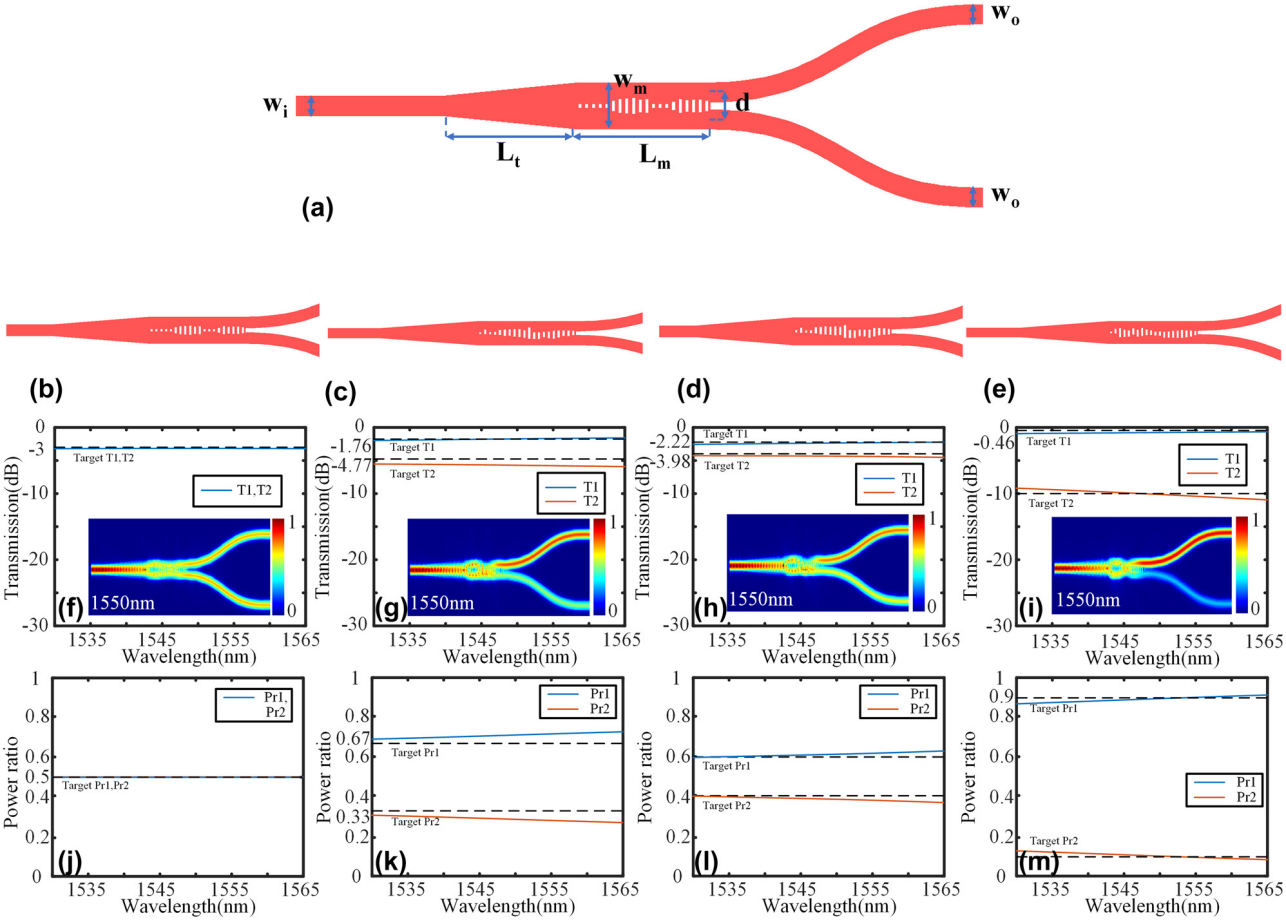
The design and simulation result of 1x2 power splitter: (a) The base structure of 1 × 2 power splitters. 1 × 2 power splitters with (b) 1:1 power ratio, (c) 2:1 power ratio, (d) 6:4 power ratio, and (e) 9:1 power ratio. Transmission spectra and the simulated electric field profiles of (f) 1:1 power splitter, (g) 2:1 power splitter, (h) 6:4 power splitter, and (i) 9:1 power splitter. The power ratio of (j) 1:1 power splitter, (k) 2:1 power splitter, (l) 6:4 power splitter, and (m) 9:1 power splitter.

**Figure 6:**
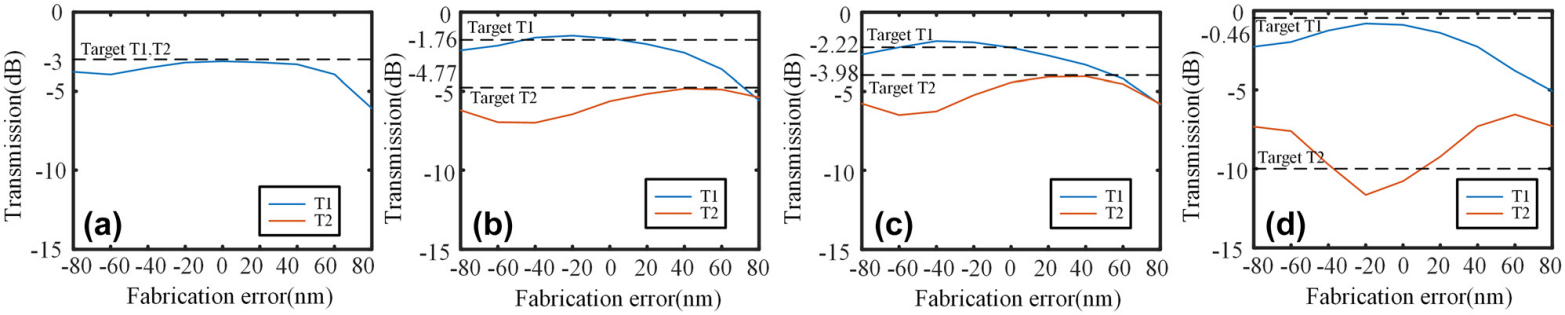
The fabrication error simulation of 1 × 2 power splitters with (a) 1:1 power ratio, (b) 2:1 power ratio, (c) 6:4 power ratio, and (d) 9:1 power ratio.

The splitters for the standard fully etched SOI platform with 220 nm Si have been designed, and the structure base is illustrated in Figure 5(a). The width of input and output ports *w*_*i*_ and *w*_o_ is 0.5 μm, and the distance between two outputs *d* is 0.7 um. The length *L*_m_ and width *w*_m_ of the mid rectangle are 4 μm and 1.2 μm. The length of the taper *L*_t_ is 4 μm. The period *Λ* of the metamaterial is 0.2 μm, and the number of periods is 20. The etched rectangle has a minimum length of 0.1 μm, with upper and lower limits set at 0.3 μm and 0.05 μm, respectively. The dataset differs for symmetry and asymmetric power ratio. Specifically, we utilize a dataset consisting of 2,000 symmetric structures and 3,000 asymmetric structures to represent the symmetric and asymmetric power ratios respectively. The RMSE of the KNN is 0.0417 and 0.0425 for symmetric and asymmetric problems, respectively. The entire optimization process takes 37.5 h (CPU: AMD Ryzen Threadripper3970X). Figure 5(b)–(e) depict the 1 × 2 power splitters achieved through KNN with varying power ratios. Figure 5(f)–(i) showcase the transmission spectra and simulated electric field profiles of the power splitters at 1550 nm with different power ratios. Figure 5(j)–(m) illustrate the power ratio. The insertion loss at 1550 nm is 0.17 dB, 0.26 dB, 0.22 dB, and 0.29 dB for 1:1, 2:1, 6:4, and 9:1 power ratios, respectively. The splitters also exhibit great operational bandwidth. The insertion loss at 1,530–1,565 nm is below 0.21 dB, 0.39 dB, 0.32 dB, and 0.32 dB for power ratios of 1:1, 2:1, 6:4, and 9:1, respectively. The similarity between the structures of 2:1 and 6:4 can be attributed to the underlying principle of KNN. Once the dataset is determined, KNN selects k values that are closest to the target result for further computation in order to obtain the desired parameters. It is worth noting that identical k values yield consistent outcomes. Given that both power ratios of 2:1 and 6:4 exhibit similarities, it can be inferred that they fall within a similar range of k values, thereby resulting in comparable results. This observation also underscores the stability inherent in the KNN method. The results of each calculation for other inverse design methods will exhibit substantial disparities. The impact of fabrication errors on the IM structure was also simulated and analyzed, as depicted in Figure 6. For a 1:1 splitter, a ±40 nm fabrication error only results in a performance degradation of 0.5 dB. In the case of a 2:1 splitter and a 6:4 splitter, the fabrication error required for a 0.5 dB performance degradation is approximately −10 – +20 nm. For the 9:1 splitter, from −40 nm to +20 nm, the performance degradation of T1 is lower than 0.5 dB, and the performance degradation of T2 is lower than 1.5 dB. It can be observed that the IM structure exhibits significant fabrication tolerance.

### 2.3 1 × 3 power splitters

**Figure 7:**
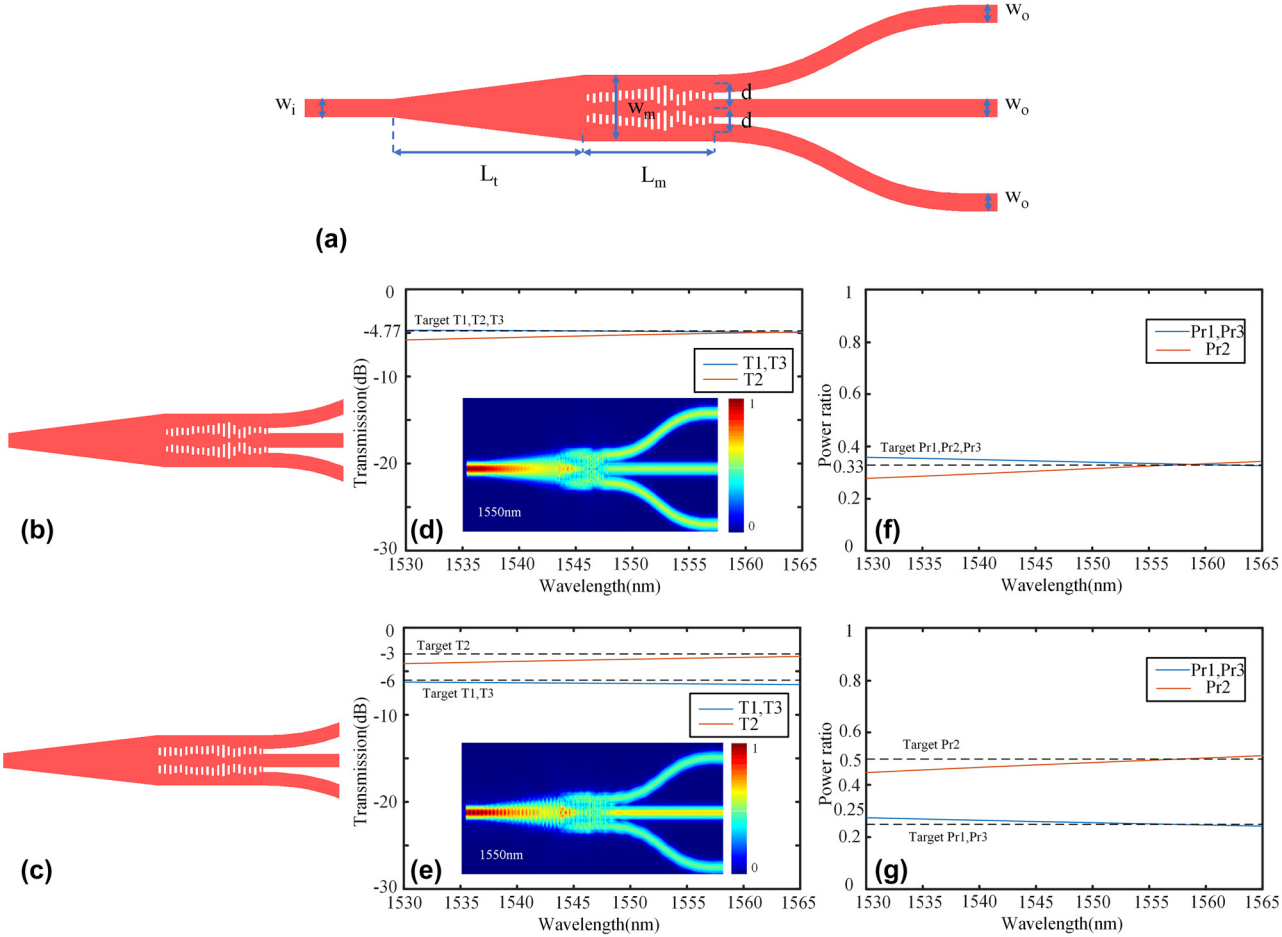
The design and simulation result of 1x3 power splitter: (a) The base structure of 1 × 3 power splitters. 1 × 3 power splitters with (b) 1:1:1 power ratio, and (c) 1:2:1 power ratio. Transmission spectra and the simulated electric field profiles of (d) 1:1:1 power splitter, and (e) 1:2:1 power splitter. Power ratio of (f) 1:1:1 power splitter, and (g) 1:2:1 power splitter.

**Figure 8:**
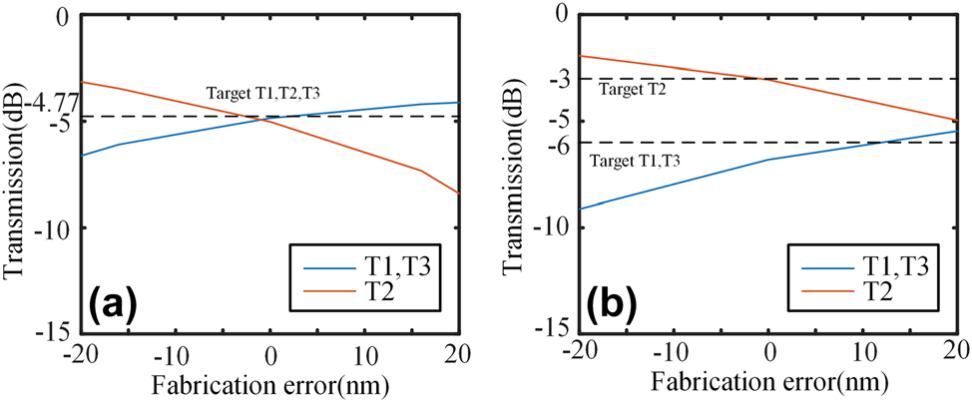
The fabrication error simulation of 1 × 3 power splitters with (a) 1:1:1 power ratio, and (b) 1:2:1 power ratio.

The feasibility of this scheme for arbitrary port numbers was demonstrated by designing 1 × 3 power splitters with arbitrary power ratios, and Figure 7(a) illustrates the base structure. The width of input and output ports *w*_i_ and *w*_o_ is 0.5 μm, and the distance between three outputs *d* is 0.7 μm. The length *L*_m_ and width *W*_m_ of the mid rectangle are 4 μm and 1.9 μm. The length of the taper *L*_t_ is 6 μm. The experiment employs two metamaterial arrays, with a center-to-center distance of 0.7 μm. The metamaterial has a period length of 0.2 μm, consisting of 20 periods. It is important to note that our dataset comprises 3,000 symmetric and 5,000 asymmetric structures. The KNN model achieves an RMSE value of 0.0394. The entire optimization process takes 22.5 h. The 1 × 3 power splitters with different power ratios obtained by KNN are shown in Figure 7(b) and (c). Simulated electric field profiles and transmission spectra of the power splitters at 1550 nm with different power ratios are shown in Figure 7(d) and (e). Power ratios are depicted in Figure 7(f) and (g). The insertion loss at 1550 nm is 0.17 dB, and 0.49 dB for 1:1:1, and 1:2:1 power ratio, respectively. The insertion loss in the entire C band (1,530–1565 nm) is below 0.27 dB and 0.65 dB for power ratios of 1:1:1 and 1:2:1 respectively. The fabrication errors of the 1 × 3 power splitters were simulated, as depicted in Figure 8. A fabrication error range of −12 – +7 nm resulted in a performance degradation lower than 0.8 dB for the 1:1:1 splitter. Similarly, for the 1:2:1 splitter, a ±7 nm fabrication error led to a performance degradation of 0.8 dB. The fabrication tolerance of these 1 × 3 power splitters is not as good as that of the 1 × 2 splitters, but it still exhibits excellent manufacturability within the existing fabrication platform.

### 2.4 1 × 4 power splitters

**Figure 9:**
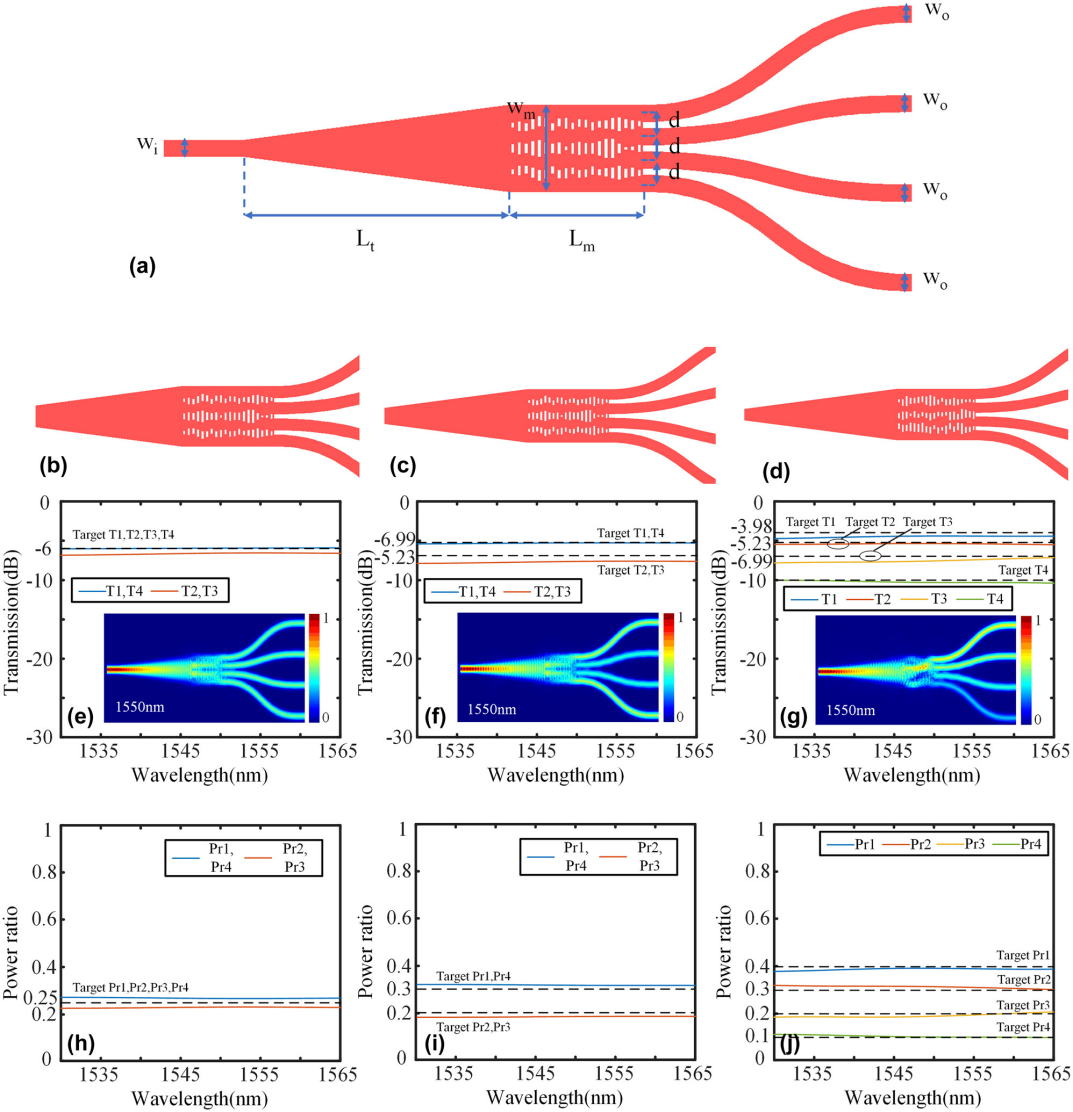
The design and simulation result of 1x4 power splitter: (a) The base structure of 1 × 4 power splitters. 1 × 2 power splitters with (b) 1:1:1:1 power ratio, (c) 3:2:2:3 power ratio, and (d) 4:3:2:1 power ratio. Transmission spectra and the simulated electric field profiles of (e) 1:1:1:1 power splitter, (f) 3:2:2:3 power splitter, and (g) 4:3:2:1 power splitter. The power ratio of h) 1:1:1:1 power splitter, (i) 3:2:2:3 power splitter, and (j) 4:3:2:1 power splitter.

**Figure 10:**
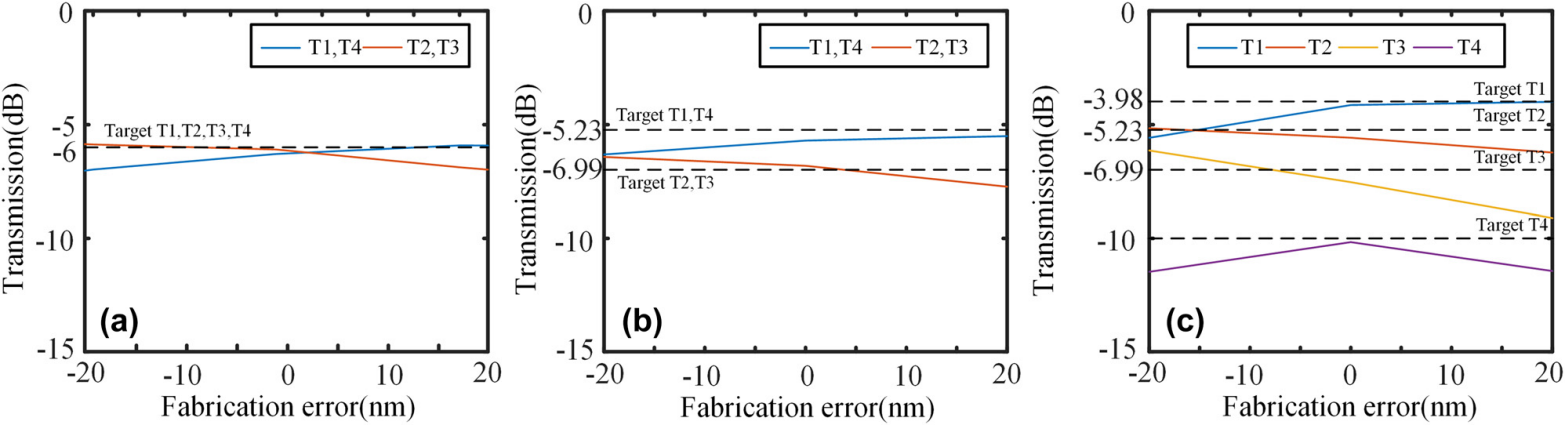
The fabrication error simulation of 1 × 4 power splitters with (a) 1:1:1:1 power ratio, (b) 3:2:2:3 power ratio, and (c) 4:3:2:1 power ratio.

Compared to 1 × 3 power splitters, 1 × 4 power splitters are more appealing and extensively researched. However, there have been limited reports on 1 × 4 power splitters with arbitrary power ratios. In this study, we have designed 1 × 4 power splitters based on the IM method. Figure 9(a) illustrates the fundamental structure of the 1 × 4 power splitter. The width of input and output ports *w*_i_ and *w*_*o*_ is 0.5 μm, and the distance between adjacent two output waveguides *d* is 0.7 μm. The length *L*_m_ and width *w*_m_ of the mid rectangle are 4 μm and 2.6 μm. The length of the taper *L*_t_ is 8 μm. We utilize three arrays of metamaterials, with a center-to-center distance between the two arrays of 0.7 μm. The metamaterial has a period length of 0.2 μm, and there are a total of 20 periods. The dataset for symmetric and asymmetric power ratios consisted of 5,000 symmetric structures and 8,000 asymmetric structures. The KNN achieved an RMSE of 0.0184 for the symmetric power ratio and 0.0243 for the asymmetric power ratio. The entire optimization process takes 97.5 h. It can be seen the increase in parameters necessitates a larger dataset for KNN to achieve optimal performance. The 1 × 4 power splitters with different power ratios obtained by KNN are shown in Figure 9(b) and (c). The transmission spectra and simulated electric field profiles of the power splitters at 1550 nm are shown in Figure 9(d) and (e) for different power ratios. Figure 9(f) and (g) displays the power ratio. The insertion loss at 1550 nm is 0.24 dB, 0.28 dB, and 0.35 dB for the power ratios of 1:1:1:1, 3:2:2:3, and 4:3:2:1, respectively. The insertion loss is lower than 0.42 dB, 0.47 dB, and 0.53 dB for power ratios of 1:1:1:1, 3:2:2:3, and 4:3:2:1, respectively, within the operation bandwidth of 1,530–1,565nm. The simulated results in Figure 10 demonstrate that a fabrication error of ±20 nm only leads to a performance degradation lower than 0.9 dB for the 1:1:1:1 power splitter. Similarly, for the 3:2:2:3 splitter, a ±20 nm fabrication error results in a performance degradation of only 0.6 dB. Furthermore, the fabrication tolerance range for the 4:3:2:1 splitter to achieve a performance of 0.8 dB is within ±10 nm. The simulation of fabrication errors serves as evidence for the excellent fabrication tolerance of the proposed structures.

## 3 Fabrication and experimental result

### 3.1 1 × 2 power splitters

**Figure 11:**
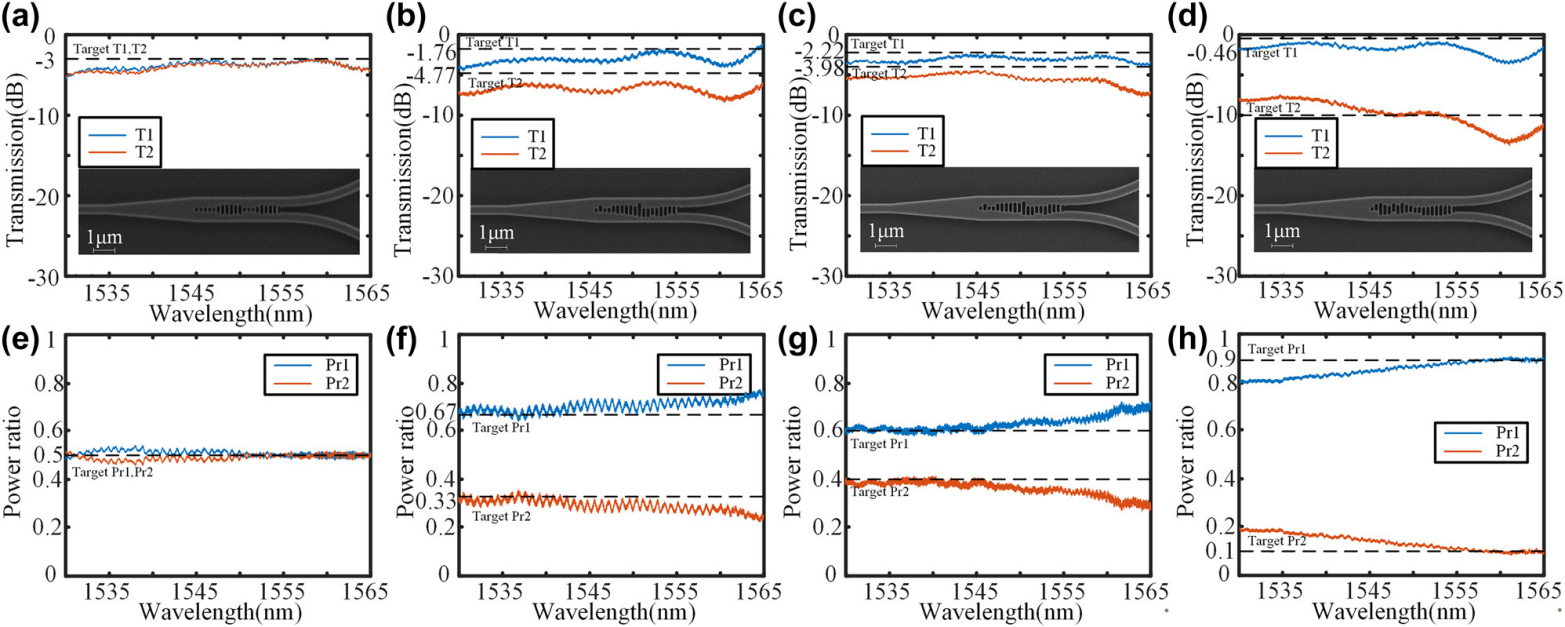
Transmission spectra and the SEM image of (a) 1:1 power splitter, (b) 2:1 power splitter, (c) 6:4 power splitter, and (d) 9:1 power splitter. Power ratio of (e) 1:1 power splitter, (f) 2:1 power splitter, (g) 6:4 power splitter, and (h) 9:1 power splitter.

The optimized structures were fabricated on the SOI platform, utilizing a silicon substrate with a thickness of 725 μm, SiO_2_ box layer of 2um, and silicon device layer of 220 nm. 100 keV electron beam lithography (EBL, Vistec EBPG5000+ES) was used to define the patterns. The wave-guide is fully etched down using inductively coupled plasma (ICP, Oxford Plasmalab System 100). In order to achieve precise manufacturing of the metamaterial region, we made a slight adjustment to the duty cycle of the subwavelength array in the layout, setting it at 40 %. Additionally, we implemented over etching treatment in ICP to ensure accurate preparation of the metamaterial region. The fabricated device is characterized using a C-band source and an optical spectrum analyzer (OSA). The continuous-wave broadband light is coupled into the input grating and received from the output grating by the OSA for transmission. Figure 11(a)–(d) show the transmission spectra and the scanning electron microscope (SEM, GeminiSEM 300) image of 1 × 2 splitters, and Figure 11(e)–(h) shows the power ratio using the second port as a reference. The subwavelength grating structure ensures excellent fabrication consistency, leading to SEN images that exhibit exceptional fabrication accuracy. The insertion loss at 1,550 nm is approximately 0.71 dB, 0.59 dB, 0.83 dB, and 0.36 dB for the ratios of 1:1, 2:1, 6:4, and 9:1, respectively. It can be observed that the power ratio closely approximates the ideal value. The reason for this is that, the experimental and simulation results exhibit excellent agreement, particularly in terms of the power ratios which demonstrate consistent behavior. This validates the efficacy of the proposed IM inverse design method for 1 × 2 power splitters with arbitrary power ratios.

### 3.2 1 × 3 power splitters

**Figure 12:**
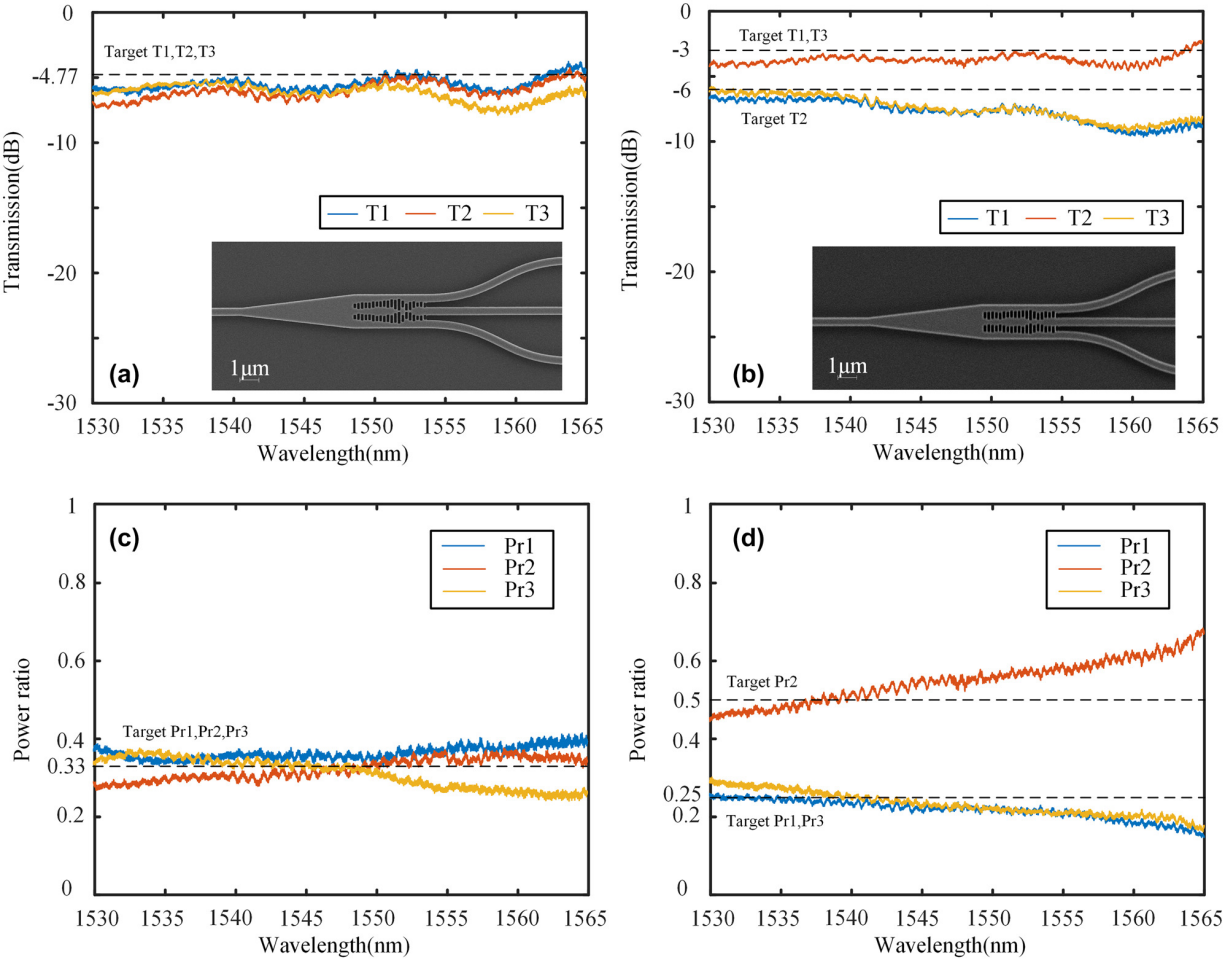
Transmission spectra and the SEM image of (a) 1:1:1 power splitter, and (b) 1:2:1 power splitter. Power ratio of (c) 1:1:1 power splitter, and (d) 1:2:1 power splitter.

The 1 × 3 power splitters were fabricated on the same SOI, and the experimental setup for the 1 × 2 power splitters remains unchanged. The transmission spectra and SEM image of 1 × 3 splitters are presented in Figure 12(a) and (b), demonstrating excellent fabrication accuracy as indicated by the SEN images. The power ratios are illustrated in Figure 12(c) and (d). In contrast to 1 × 2 power splitters, which have two ports, 1 × 3 power splitters possess three ports. There-fore, we designate the third port as the reference for measuring the power ratios. The insertion loss at 1,550 nm is 0.54 dB and 0.95 dB for the configurations of 1:1:1 and 1:2:1, respectively. The results of the 1:1:1 splitter are consistent when compared to the simulation results. However, for the 1:2:1 splitter, there is a slight deviation in power ratio due to a decrease in T1 and T3 at long wavelengths. The performance degradation can be attributed to the inconsistency in the grating coupler’s performance, which falls within the acceptable error range. The experimental results demon-strate the scheme’s feasibility for multi-ports with arbitrary power ratios and validate its scalability in designing various structural devices.

### 3.3 1 × 4 power splitters

**Figure 13:**
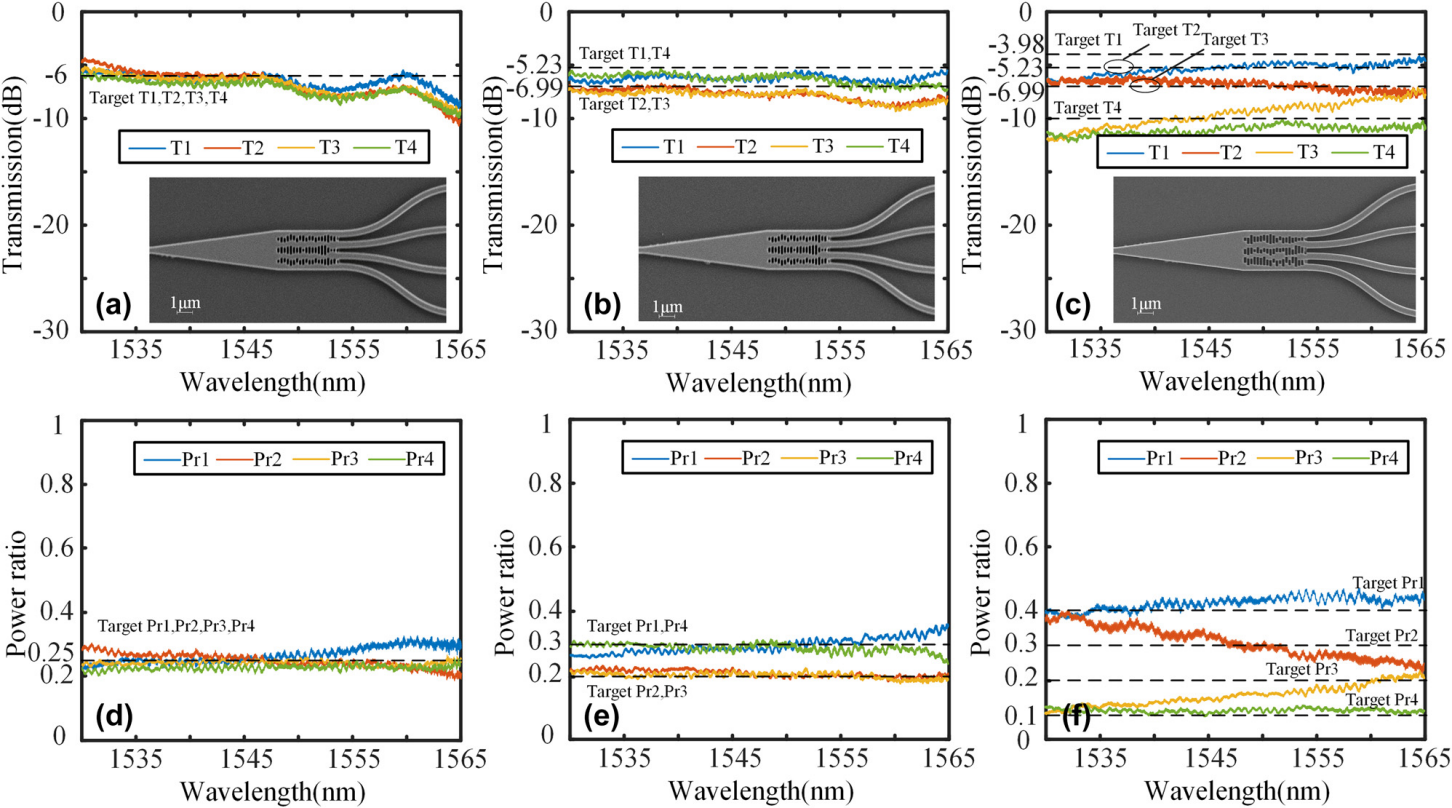
Transmission spectra and the SEM image of (a) 1:1:1:1 power splitter, (b) 3:2:2:3 power splitter, and (c) 4:3:2:1 power splitter. Power ratio of (d) 1:1:1:1 power splitter, (e) 3:2:2:3 power splitter, and (f) 4:3:2:1 power splitter.

We also fabricated 1 × 4 power splitters on the same substrate, and Figure 13(a)–(c) displays the transmission spectra and SEM image of 1 × 4 splitters. The advantage of our IM method is that the fabrication difficulty does not increase with the complexity of the structure, and the SEN images still exhibit consistent fabrication quality. The power ratio is illustrated in Figure 13(d)–(f) with the fourth port serving as a reference. The insertion loss at 1,550 nm is approximately 0.98 dB, 0.85 dB, and 0.95 dB for the configurations of 1:1:1:1, 3:2:2:3, and 4:3:2:1, respectively. The performance of both the 1:1:1:1 splitter and the 3:2:2:3 splitter is highly consistent with the simulation. However, it can be observed that the performance of the 4:3:2:1 splitter is significantly affected by changes in wavelength. The fabrication error significantly affects the control of such a precise power ratio due to the presence of numerous small structures. The performance at a specific wavelength still demonstrates the solution’s feasibility. In general, the 1 × 4 splitters demonstrate the potential and feasibility of the IM method for complex design.

### 3.4 Transmission experiment

**Figure 14:**
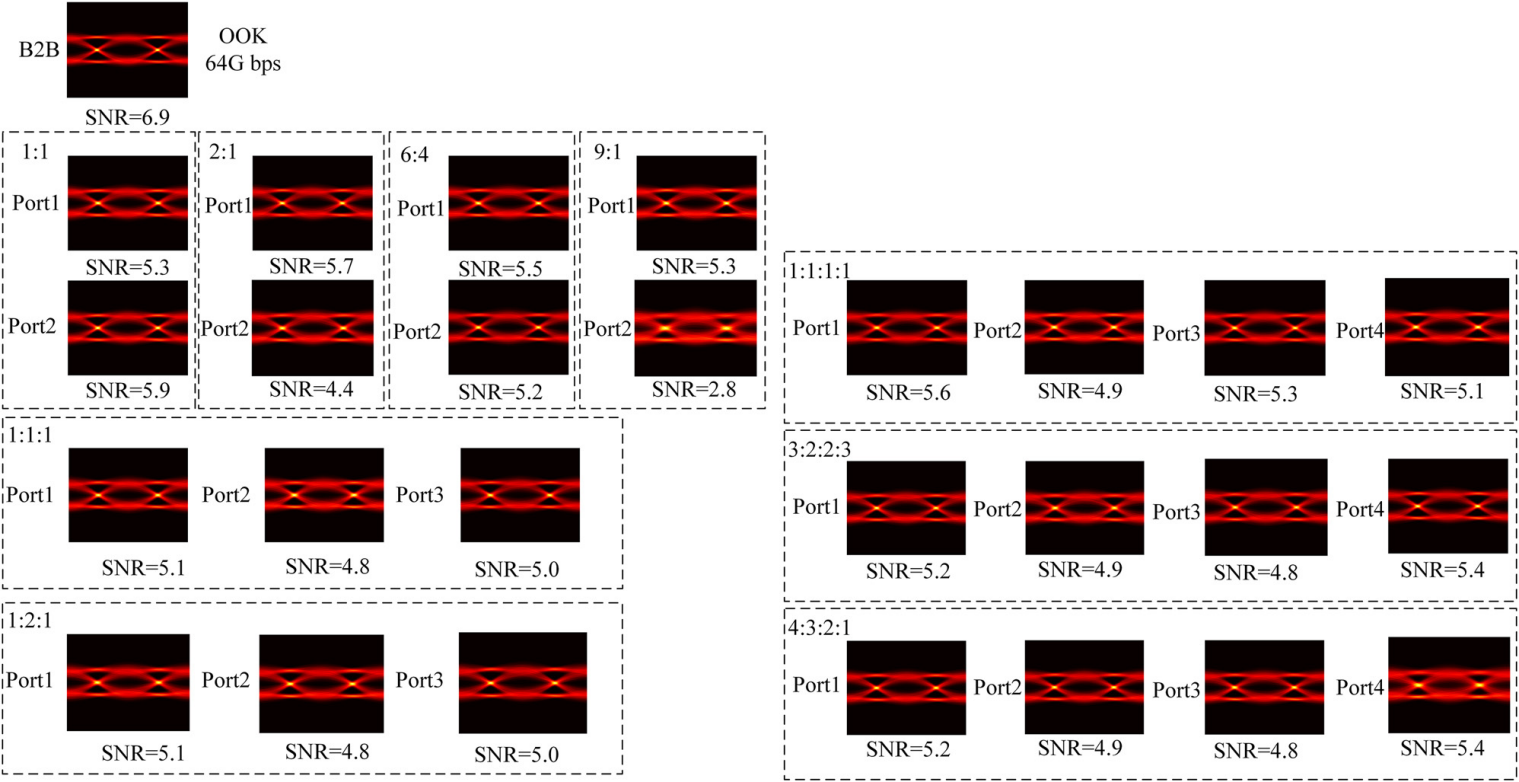
Eye diagrams and signal-to-noise ratios of the 64 Gbps OOK signal transmission.

To verify the signal transmission performance of our devices, a high-speed transmission experiment is performed. An OOK signal with 64 Gbit/s is used. An ECL laser with a wavelength of 1,550 nm and linewidth of 100 kHz is used as the optical signal carrier. An arbitrary waveform generator (AWG, Keysight M8195A) is used to generate the electrical signal. The signal is modulated by a commercial LiNbO3 Mach−Zehnder modulator (MZM, JDSU X5). The detected electrical signals are captured by a 100-GSa/s digital sampling oscilloscope (DSO, Tektronix DPO73304D). Figure 14 shows the received eye diagrams and signal-to-noise ratio (SNR) of all ports. We can observe the open and clear eye diagrams. The degradation is mainly caused by insertion loss in transmission, thus port2 of 9:1 and port4 of 4:3:2:1 have more degradation than others.

## 4 Comparison

We compare the insertion loss, structure length, and scalability of the proposed device with reported experimentally demonstrated arbitrary ratio power splitters on SOI. As shown in Table 1, we can see the reported structure can be divided into traditional structure and inverse design structure. For traditional structure, the footprint is usually large, and the scalability is low. When the number of output ports increases, inverse engineering devices have significant expansion advantages. Moreover, device in this paper has no holes and cracks compared to DBS or shape-optimization structure, which reduce fabrication difficulties. Based on the previous discussion on computational complexity, this method has significant advantages in inverse design structures.

**Table 1:** Summary of experimentally demonstrated arbitrary ratio power splitters on SOI.

Device	Method	IL (dB)	Length (μm)	Scalability
Traditional structure	DC [20]	<0.22	79	1 × 2 (low)
	SWG DC [21]	<1	11.5	1 × 2 (low)
	SWG Y-junction [ [22]	<0.82	2.9	1 × 2 (low)
	MMI [23]	<1.2	75	1 × 2 (low)
Inverse design structure	Shape optimization [24]	<0.54	14	1 × 2 (high)
	DBS [25]	∼ 1	3.6	1 × 2,1 × 3 (high)
	This work	<1	<4	1 × 2, 1 × 3, 1 × 4 (high)

Based on the 1 × 2 structure, we also compared the neural network method (TNN) [13] with the KNN method in this paper and found that KNN performed much better than TNN on the same 5,000 dataset. The performance of TNN approaches that of KNN when the dataset size reaches 60,000. However, considering the excessive number of datasets required by TNN compared to what KNN needs, it is evident that KNN holds significant advantages under the condition of a small dataset size.

## 5 Conclusions

In this paper, we propose an intelligent metamaterial structure, which is enhanced by the integration of PSO and KNN algorithms, enabling rapid acquisition of diverse outputs within the same structural type. The intelligent meta-material combines compactness and design flexibility while avoiding the fabrication challenges of some inverse design devices. Compared to commonly used neural network methods, KNN is simpler and requires less data volume. By utilizing this approach, we successfully designed and manufactured power splitters with varying power ratios, including 1 × 2, 1 × 3, and 1 × 4 configurations. The obtained results validate the effectiveness of this method in designing splitters with any desired number of ports and power distribution ratios. Moreover, thanks to the IM structure and design methodology employed here, we can extend our design capabilities to encompass multimode splitters as well as other functionalities beyond just power splitting. The method proposed here offers a fresh perspective on the inverse design of photonics.
